# Pupil responses associated with the perception of gravitational vertical under directional optic flows

**DOI:** 10.1038/s41598-021-00346-y

**Published:** 2021-10-29

**Authors:** Joo Hyun Park, Sung Ik Cho, June Choi, JungHyun Han, Yoon Chan Rah

**Affiliations:** 1 Department of Otorhinolaryngology-Head and Neck Surgery, Dongguk University College of Medicine, Ilsan Hospital, Goyang, Republic of Korea; 2 Department of Computer Science and Engineering, Korea University College of Informatics, Seoul, Republic of Korea; 3 Department of Otorhinolaryngology-Head and Neck Surgery, Korea University Ansan Hospital, Korea University College of Medicine, Seoul, Republic of Korea

**Keywords:** Experimental models of disease, Preclinical research, Clinical trial design

## Abstract

This study assessed the pupil responses in the sensory integration of various directional optic flows during the perception of gravitational vertical. A total of 30 healthy participants were enrolled with normal responses to conventional subjective visual vertical (SVV) which was determined by measuring the difference (error angles) between the luminous line adjusted by the participants and the true vertical. SVV was performed under various types of rotational (5°/s, 10°/s, and 50°/s) and straight (5°/s and 10°/s) optic flows presented via a head-mounted display. Error angles (°) of the SVV and changes in pupil diameters (mm) were measured to evaluate the changes in the visually assessed subjective verticality and related cognitive demands. Significantly larger error angles were measured under rotational optic flows than under straight flows (*p* < 0.001). The error angles also significantly increased as the velocity of the rotational optic flow increased. The pupil diameter increased after starting the test, demonstrating the largest diameter during the final fine-tuning around the vertical. Significantly larger pupil changes were identified under rotational flows than in straight flows. Pupil changes were significantly correlated with error angles and the visual analog scale representing subjective difficulties during each test. These results suggest increased pupil changes for integrating more challenging visual sensory inputs in the process of gravity perception.

## Introduction

Accurate estimation of the direction of gravity is essential for spatial orientation and postural stability. Inputs from various sensory systems, including the otolith organs, visual information, and proprioception, are centrally integrated to generate an internal estimate of the direction of gravity^1–3^. In addition to the information from the otolith organs, accurate visual information plays an important role in calculating the internal verticality. In other words, confusing or limited visual information could induce a failed perception of the accurate direction of the gravitational vertical, which can be observed in several cases of air crashes from limited visual reference during starless night flight through a thick cloud or flying over featureless terrain^4–6^.

Experimentally, the presence of additional visual cues can influence the accuracy of subjectively perceived verticality^7^. In particular, the rotating optokinetic stimulus could shift the subjectively perceived vertical direction^7–11^. Ocular torsion induced by the rotating optokinetic flow has been suggested as an underlying physiologic mechanism that biases the perception of verticality, especially when the subjective verticality was assessed in a visually controlled way, such as the subjective visual vertical (SVV)^12–15^. However, the underlying mechanisms of this optokinetic stimulation-induced bias of SVV are not fully understood.

As such, the determination of subjective verticality could require a considerable amount of cognitive resources as the process has to evaluate and integrate multiple sensory information. In other words, the process could be easily affected by conflicting or confusing sensory information, especially for those with limited cognitive reserve. Previous studies have reported the role of higher cortical functions as well as brainstem reflexes in the internal estimation of the direction of gravity, possibly confirming the potential role of cognitive functions^10,16^. Reportedly, age-associated increasing dependence on visual cues might suggest an increased probability of failed central integration of multiple sensory information^17^. The pupil response has been used to measure the required cognitive load or attention in a variety of task types^18–21^. In general, pupil dilation can be promptly observed following the onset of relevant stimuli with an increase in the cognitive demands of a task^18–21^. Among the clinical tests for vestibular functions, pupil response can be preferentially applied to the SVV because it is performed in a dark visual field with minimal eye movement, making it easy to reliably measure the pupil size.

In this study, we aimed to evaluate the cognitive demands required to integrate confusing optokinetic stimuli during the subjective perception of verticality by assessing pupil changes. The association between the accuracy of verticality perception and cognitive demands was also analyzed.

## Methods

### Participants

A total of 30 healthy participants (male:female = 8:22, an average age of 38.5 y with a range of 24–62 y) without any history of neuro-otologic or vestibular disorders were enrolled in the study. All the participants reported near-normal visual acuity of the naked eye (better than 20/25 of the Snellen chart in the better eye without anisometropia). Those with visual, psychological, systemic disease, and medications that could affect the equilibrium or pupil responses were excluded, and those weighing more than 90 kg were also excluded owing to restrictions in equipment usage. The participants underwent conventional SVV tests (JI SVV, Jeil Inc., Republic of Korea) prior to participating in the experiments for ruling out potential dysfunction related to the perception of the gravitational vertical. All the participants had normal SVV findings, defined as error angles less than 2°. The measurements were performed at the Korea University Ansan Hospital between March 5, 2020 and June 4, 2020. The institutional ethics committee approval was obtained from the Korea University Ansan Hospital (2020AS0057). All methods were performed in accordance with the relevant guidelines and regulations. Informed consent was obtained from all the participants.

### Stimuli and equipment

Directional optic flows were generated by moving multiple tiny dot patterns in the programmed directions and velocities. They were presented through a head-mounted display (HMD) (Vive Pro Eye, HTC, Taiwan). The visual stimuli were constructed by creating a large virtual spherical space with a radius of 10 m from the participant’s point of view. The background moving objects (0.125 m of virtual radius) were created on the surface of the sphere with a density of 0.55 objects/m^2^. The field of view was a small area of the virtual sphere with a visual angle of 10° on latitude and longitude centered on the equator of the sphere.

To measure the perceived gravitational vertical, an adjustable rotating test rod was placed in the center of the screen and controlled in the CW and CCW directions at a speed of 5°/s (continuously) or with 0.1°/s (per click) by a controller (Vive Pro Eye, HTC, Taiwan). The participant was instructed to align the test rod in the direction of the subjectively perceived direction of gravitational vertical from the initially tilted offset at 30°or 330°. The error angle to the actual gravitational vertical was calculated with a resolution of 10^–6^° in the roll plane for each test.

The pupil diameter was measured for each test using an eye tracker (incorporated within Vive Pro Eye, HTC, Taiwan). A dark visual field was obtained by tightly fitting the HMD to the face in a dark experimental room with the lights turned off. The luminance of the test screen was measured for each experiment by calculating the L* value of the CIE-Lab color space and luminance (Y) value of YIQ color space^22^. These color components were transformed from the originally extracted sRGB component of each test screen. The L* of CIE-Lab, which ranges from 0 (dark) to 100 (light), reflects the lightness felt by humans, independent of the medium and the Y component of the YIQ color space represents the luminance in a computer image^23,24^.

### Procedures and measured data

The participants sported an HMD with a controller in their preferred hand, which was established on a force plate (i2a systems, Republic of Korea). Two inertial measurement units (IMUs) were placed in the center of gravity (CoG) and ankle (GSPI, Republic of Korea).

Three practice rounds were conducted for each experiment before the actual measurement to familiarize the participants with the program and equipment. Thirty seconds of a self-playing test screen with the same velocity of the optic flow was presented prior to each trial to revert the pupil diameter to baseline. After four seconds following the commencement of the recording, the participants were asked to align the test rod with the subjectively perceived visual vertical. A one-minute break was provided between each experiment to reduce cybersickness and eyestrain caused by repeated measurements.

**Figure 1:**
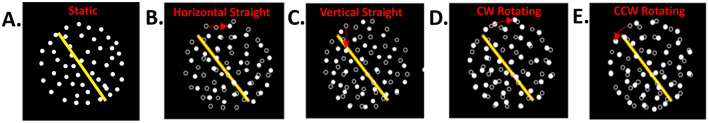
Visual stimulations. Visual stimulations consisted of one static visual stimulation and four directional structured optic flow. Static visual stimulation with the same background dot patterns of directional optic flow (**A**). Straight optic flow (5°/s, 10°/s) of horizontal (**B**), Vertical (**C**). Rotating optic flow (5°/s, 10°/s, 50°/s) in roll plane for clockwise (CW) direction (**D**), for counter-clockwise (CCW) direction (**E**).

The measurements were carried out in the following sequence of optic flows with respect to the starting offset of 330 degrees, and then another set of measurements with starting offset of 30 degrees were followed: (1) a static screen displayed with multiple motionless dot patterns (Fig. 1A). (2) straight flow in the horizontal direction (5°/s, 10°/s, Fig. 1B), (3) straight flow in the gravitational vertical direction (5°/s, 10°/s, Fig. 1C), (4) clockwise (CW, Fig. 1D) rotational flows, and (5) counter-clockwise (CCW, Fig. 1E) rotational flows in the roll plane (5°/s, 10°/s, and 50°/s). Detailed illustrations are presented in Fig. 1.

Changes in pupil diameter were determined by calculating the difference between the diameter of maximal dilatation and baseline. A 10-Hz low-pass filter was applied to the raw data of pupil size, and linear interpolation was applied for missing data from brisk changes in pupil size. The diameter of maximal dilatation was defined by the average pupil diameter across 0.3 s of maximal dilatation, which satisfies less than 0.05 mm changes over 80% of the time interval. The baseline diameter was also defined as the average pupil diameter within a standard deviation across 4 s following the start of each measurement.

Postural sway was also measured to assess the resultant postural imbalance, especially induced by challenging stimulation that exceeds individual cognitive reserve. Three-dimensional postural sway at the CoG was obtained using the attached IMU. The maximal and average sway angles (°) were calculated from the reference point and compared between each experiment. The sway angle was automatically calculated by integrating the measured angular acceleration using the programmed algorithms. Two-dimensional postural sway of the center of pressure (CoP) was also obtained using a force plate in the X- (back-forth) and Y- (right-left) axes. The maximal and average sways (mm) were calculated from the reference point and compared between each experiment.

To quantify subjective dizziness and task-related discomfort induced by virtual reality-based visual stimulation, the dizziness handicap inventory (DHI)^25,26^, simulator sickness questionnaire (SSQ)^27^, and motion sickness susceptibility questionnaire (MSSQ)^28^ were obtained prior to the experiments and after completing the entire experiment. To briefly check the subjective dizziness caused by each experimental unit, a 10-point visual analog scale (VAS)^29^ was obtained immediately after completing each experiment.

### Comparison and statistical analysis

The error angle was a primary variable reflecting the accuracy of the participants’ perceived gravitational vertical. The error angles were compared between various experimental conditions, including different types of optic flow (static screen, straight, and rotational optic flow), different velocities of optic flow (5°/s, 10°/s, and 50°/s), and the relative direction of the test rod alignment to the background optic flow. Pupil diameter was the primary variable assessing task-related cognitive workload in each experiment. The pupil diameter was also compared between the above-mentioned experimental conditions, including different types of optic flow, different velocities of optic flow, and the relative direction of optic flow. Standardized changes in the pupil diameter, calculated by dividing the changes by baseline pupil diameter, were also evaluated. The correlation was also evaluated between error angle and pupil diameter, and pupil diameter and the results of questionnaires assessing subjective dizziness and discomfort. Postural sway was compared between various experimental conditions based on the sway angle (°) for IMU data and sway distance (mm) for force plate data.

All data are expressed as mean ± standard deviation. A paired sample t-test and one-way analysis of variance (ANOVA) with Tukey’s post-hoc test were comprehensively applied to compare the average. Error angles and pupil diameter under the same velocity of straight optic flows (horizontal, and vertical) were compared, and these were also evaluated under different velocities (5°/s, 10°/s, and 50°/s) of the same optic flow. Pearson’s correlation analysis was implemented to predict the association between the variables. Statistical analyses were performed using the SPSS software (ver. 15, SPSS Inc. Chicago, IL, USA).

## Results

### Subjective gravitational vertical under directional optic flow

All the patients underwent conventional SVV test, and all the patients recorded error angles of a normal range (< 2°) with an average of 0.49 ± 0.35°, ranging from 0.02 to 1.38°. In the subjective visual horizontal (SVH), the average error angle was 0.39 ± 0.20°, ranging from 0.03° to 0.80°.

Although the error angles were below 2° in all the measurements, significantly larger error angles were measured under a static visual field with numerous small dots in the background (SVV, 1.32 ± 1.27°) compared to the values measured with conventional SVV (0.49 ± 0.35°).

**Figure 2:**
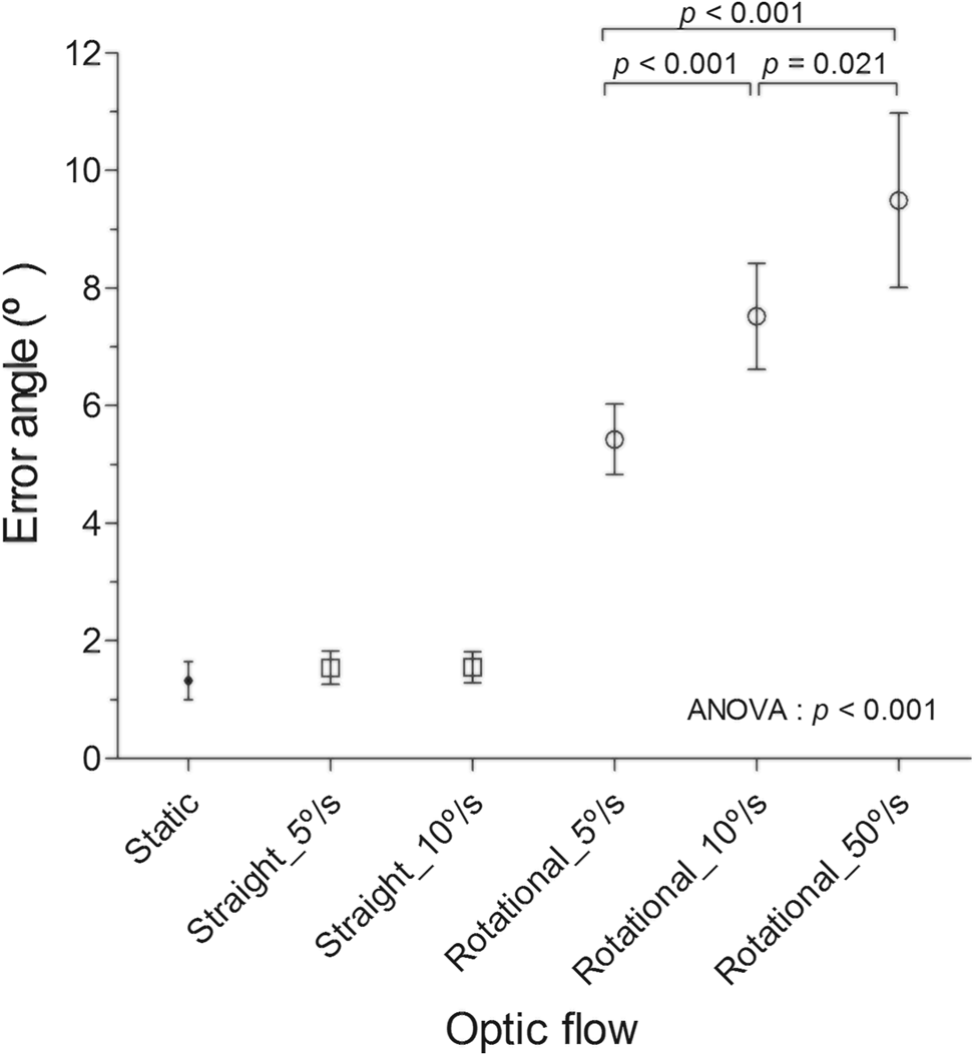
Error angles (°) for each directional optic flow (mean with 95% Confidence Intervals). Significantly higher error angles were confirmed in the rotational optic flow compared with the straight optic flow.

Compared to the mean error angles in the static background screen, those of rotational optic flows were significantly increased (*p* < 0.001 for all comparisons, Table 1A). The mean error angles of straight optic flows were increased; however, they did not differ from those of the static screen and were below 2°. The error angles of each test condition were also compared with those of the straight optic flow with the slowest velocity (5°/s), and the error angles of the rotational optic flows were confirmed to be significantly higher (*p* < 0.001 for all comparisons, Table 1B). The error angles also significantly increased as the angular velocity increased for rotatory optic flows (Fig. 2 and Table 1C). For straight optic flows, the error angles were not significantly different for the different directions of optic flow (horizontal and vertical flow) and for the different velocities of the optic flow (Supplementary Table 1A and 1B). The error angles of SVV were larger when the direction of the test rod adjustment was aligned in the opposite direction (tilt offset of 30° under CW rotatory stimuli and tilt offset of 330° under CCW rotational stimuli) for 10°/s rotational optic flows than in the same direction (tilt offset of 30° under CCW rotational stimuli and tilt offset of 330° under CW rotatory stimuli) (*p* = 0.492, t = 0.691, df = 62 for 5°/s and *p* = 0.008, t = 2.703, df = 62 for 10°/s). The details are shown in Supplementary Table 1B.

**Table 1:** Comparison of the error angles of each experimental condition of optic flows.

Direction 1	Direction 2	Velocity (°/s)	Error angles (°)	*p-*value	*t*	*df*		
**A. Comparison with the error angles of the static background screen**								
Static 1.32 ± 1.27°	Straight	5	1.54 ± 1.60	0.162	1.405	119		
		10	1.55 ± 1.49	0.116	1.580	119		
	Rotational	5	5.42 ± 3.37	< 0.001*	13.23	119		
		10	7.52 ± 5.08	< 0.001*	14.39	119		
		50	9.49 ± 5.54	< 0.001*	11.08	59		
**B. Comparison with the error angles of the straight 5°/s (horizontal 5°/s + vertical 5°/s)**								
Straight 5°/s 1.54 ± 1.60°	Straight	10	1.55 ± 1.49	0.9260	0.093	119		
	Rotational	5	5.42 ± 3.37	< 0.001*	12.14	119		
		10	7.52 ± 5.08	< 0.001*	13.36	119		
		50	9.49 ± 5.54	< 0.001*	10.65	59		

Type of optic flow	Velocity of optic flow					Post hoc analysis (p)		
	5°/s	10°/s	50°/s	*p*	F	5–10	5–50	10–50
**C. Comparison of the error angles of different velocities for the rotational optic flow**								
Rotational	5.42 ± 3.37	7.52 ± 5.08	9.49 ± 5.54	< 0.001*	16.51	0.001*	< 0.001*	0.021*

### Pupil responses

**Figure 3:**
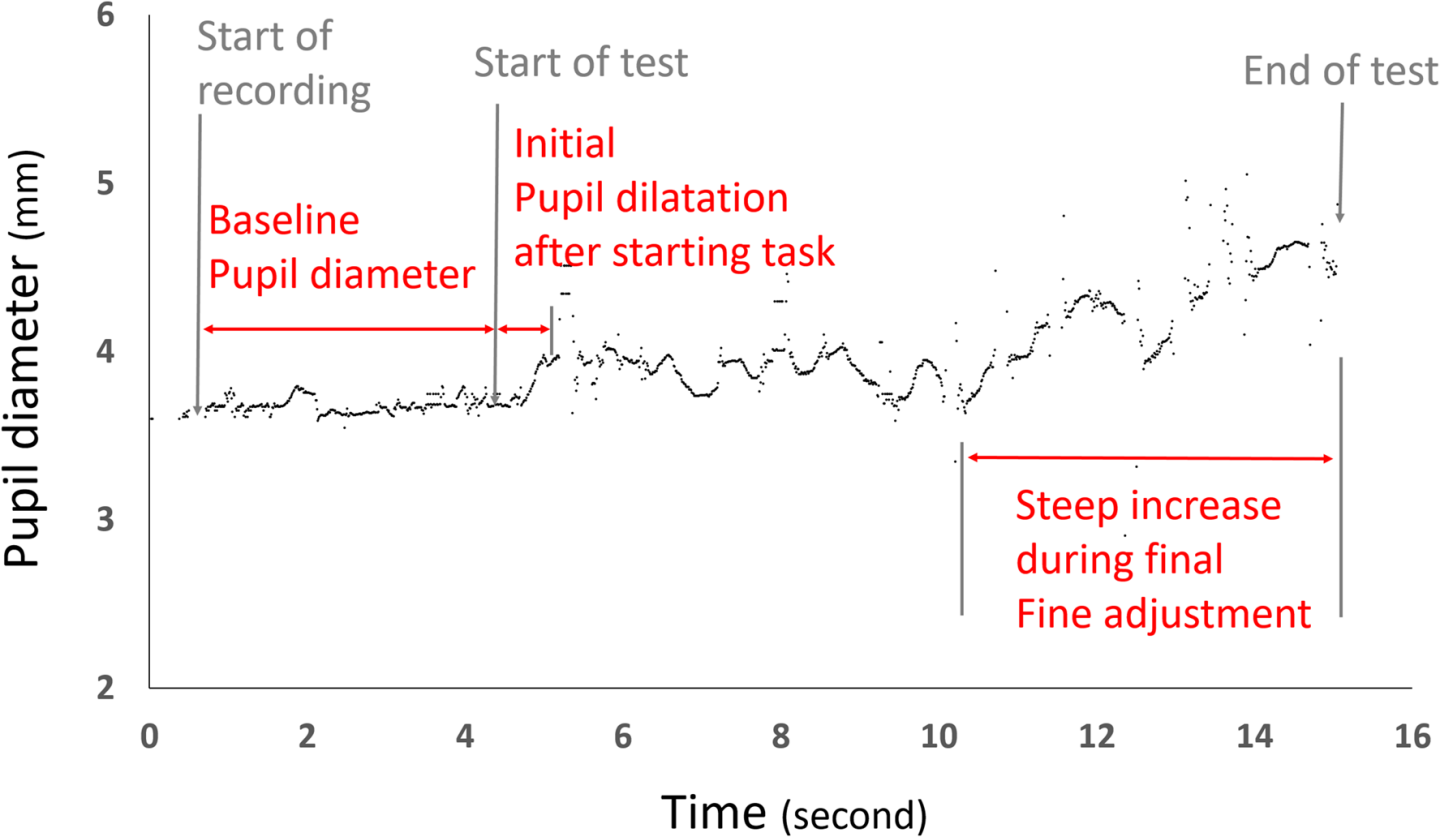
Examples of pupil response during measurement (from case #3, under clockwise rotational flow of 10°/s). Baseline pupil diameter was measured approximately 4 s prior to starting the test. Initial pupil dilatation was recorded after starting the test rod adjustment. Significant increase of the pupil diameter was observed when finely aligning the bar near the vertical.

In general, the pupil diameter increased following the start of the experiment and an additional increase in the pupil diameter was observed during the final fine alignment of the test rod to the gravitational vertical direction (Fig. 3).

**Figure 4:**
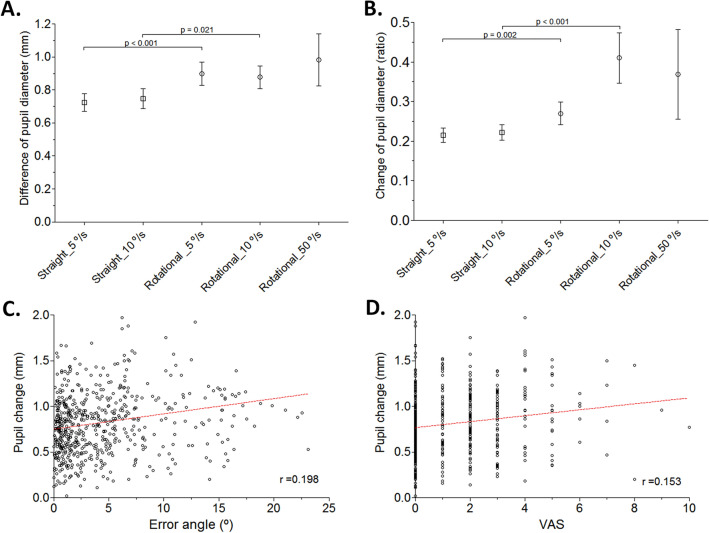
Changes (mm, **A**) and standardized changes (ratio, **B**) relative to baseline in the pupil diameter under visual stimulation and relationship between the pupil change and error angle (**C**), and visual analog scale (VAS) of subjective dizziness (**D**). Significantly larger changes in the pupil diameter was observed under rotational stimulation (mean with 95% confidence intervals). Significant correlations were found between error angle and the changes in pupil diameter (*p* = 0.004) and VAS for each test condition and the changes in pupil diameter (*p* = 0.005).

Significantly larger increases in the pupil size were confirmed for rotational optic flows compared to the pupil changes of the static screen, whereas the changes were not significant for straight flows (Fig. 4A, B, Table 2A, and Supplementary Table 2A). The pupil changes in each test condition were also compared with those of the straight optic flow with the slowest velocity (5°/s), and the pupil changes of the rotational optic flows were confirmed to be significantly higher (*p* < 0.001* for 5°/s and 10°/s, *p* = 0.004* for 50°/s, Table 2B). However, the pupil changes were not significantly different according to the velocity of the straight and rotational optic flows (Table 2B, C), although the changes in the 50°/s velocity were the highest. Interestingly, for rotational flows, the pupil changes were larger when the test rod was aligned in the opposite direction (tilt offset of 30° under CW rotatory stimuli and tilt offset of 330° under CCW rotational stimuli) for the 5°/s rotational optic flows than in the same direction (tilt offset of 30° under CCW rotatory stimuli and tilt offset of 330° under CW rotational stimuli) (*p* = 0.045*, t = 2.049, df = 59 for 5°/s, *p* = 0.092, t = 1.528, df = 59 for 10°/s, Supplementary table 2B). The illuminance of the test screen showed a minimal difference between each individual measurement, without any statistical significance (*p* = 0.293 for Y component of the YIQ color space and *p* = 0.367 for L* of CIE-Lab). Detailed values are shown in Supplementary Table 3.

**Table 2:** Comparison of the pupil changes of each experimental optic flow conditions.

Direction 1	Direction 2	Velocity (°/s)	Pupil changes (mm)	*P*-value	*t*	*df*		
**A. Comparison with the pupil changes of the static background screen**								
Static Pupil changes (mm) 0.77 ± 0.36	Straight	5	0.72 ± 0.30	0.132	1.515	119		
		10	0.74 ± 0.32	0.329	0.991	118		
	Rotational	5	0.89 ± 0.38	0.007*	2.732	118		
		10	0.68 ± 0.23	0.008*	2.698	119		
		50	0.98 ± 0.58	0.019*	2.941	59		
**B. Comparison with the pupil changes of the straight 5°/s (horizontal 5°/s + vertical 5°/s)**								
Straight 5º/s Pupil changes (mm) 0.72 ± 0.30	Straight	10	0.74 ± 0.32	0.364	0.911	118		
	Rotational	5	0.89 ± 0.38	< 0.001*	6.037	118		
		10	0.87 ± 0.38	< 0.001*	4.668	119		
		50	0.98 ± 0.58	0.004*	2.941	59		

Type of optic flow	Velocity of optic flow					Post hoc analysis (*p*)		
	5°/s	10°/s	50°/s	*P-value*	F	5–10	5–50	10–50
**C. Comparison of pupil changes (mm) at different velocities for rotational optic flow**								
Rotational	0.89 ± 0.38	0.87 ± 0.38	0.98 ± 0.58	0.317	1.154	0.929	0.451	0.293

A significant correlation was found between the change in the pupil size and error angle of each test (r = 0.198, *p* = 0.004, Fig. 4C). A significant correlation was also found between the changes in pupil size and the VAS (r = 0.153, *p* = 0.005, Fig. 4D).

### Postural sways and subjective dizziness

The average sway was 5.16 ± 12.21 mm on the X- (back–forth) and 11.04 ± 19.31 mm on the Y- (right-left) axis in static visual stimulation. The postural sway was the largest in the condition of 50°/s rotational flows on the X-axis (9.83 ± 27.40) and in the 10°/s rotational flows on the Y-axis (16.25 ± 41.51); however, there was no significant statistical difference among the conditions with different optic flows. The average three-dimensional postural sway was 1.52 ± 3.48°, 1.38 ± 3.16°, and 0.06 ± 0.50° on the roll, pitch, and yaw planes, respectively. These did not differ across the test conditions, suggesting that the current test procedures had little impact on the postural stability (Supplementary Figure 1).

Surveys for subjective dizziness (DHI) and virtual reality-related dizziness (MSSQ, SSQ) were conducted, and all the scores were nearly the same before and after the measurement. The DHI was 1.37 ± 3.96 initially and changed to 1.43 ± 2.96 following the measurement (*p* = 0.532). After the measurement, the MSSQ score changed from 20.43 ± 11.19 to 19.70 ± 11.89 (*p* = 0.596) and the SSQ score changed from 6.17 ± 4.85 to 5.66 ± 5.31 (*p* = 0.845). Minimal changes in these scores suggest negligible dizziness from the virtual reality-based entire measurement procedures.

## Discussion

### Verticality

Our data confirmed significantly larger pupil dilatations along with significantly larger error angles when performing SVV under rotational optic flows in a roll plane than those under straight optic flows. Normal postural control, including the perception of verticality, requires proper integration of vestibular, visual, and somatosensory information. The relative weightings on each sensory cue vary according to the integrity of sensory information and the sensory processing ability based on personal experience and cognitive function^29–32^. Generally, limited or moving visual surrounds could deliver confusing visual information. In response, individuals reassess the changed nature of sensory cues, and adaptive reweighting is followed to determine reliable sensory cues.

SVV is a commonly practiced clinical test to measure the accuracy of subjectively estimated verticality by calculating the error angles to the gravitational vertical by adjusting a visualized test rod in the roll plane. When SVV is performed in a dark visual environment, the vestibular and somatosensory systems dominate SVV performance^7^. However, such condition is rarely experienced in the real world, and visual information cueing the direction of gravitational vertical plays an important role in the estimation of subjective verticality^1,3^. Thus, the authors attempted to evaluate the potential effect of various moving visual information in the determination of subjective verticality. The cognitive workload required in the central processing of these multiple sensory inputs was also evaluated by measuring the pupil response. Therefore, simplified moving visual stimulations were generated by altering the direction and velocity of the numerous tiny dot patterns in the black background. A dark visual field was constructed to enhance the amplitude of the pupil response. The luminance of the each test screen was also strictly controlled to minimize any potential influence of luminance on pupil response. The luminance was measured by a calculating algorithm using Y component of the YIQ color space and the lightness value (L*) of CIE-Lab. The difference in luminance was minimal, without significant differences among individual test screens.

Early pioneering research on this topic employed similar test paradigms. A tilted frame (rod and frame test)^33^ or rotating disks (rod and disk test)^9,34,35^ were developed to provide distorted visual information. Higher error angles were commonly found under a rotating visual surround for both normal participants and participants with vestibular dysfunction. The error angles were also larger for individuals experiencing persistent dizziness following vestibular neuritis^36^. Increased error angles were also observed in patients with a higher chance of visual vertigo, along with vestibular impairment^36^.

Several hypotheses have been suggested for the underlying mechanisms of the increased error angles under rotational optokinetic flows. Previously, it has been observed that a rotating optokinetic stimulus shifts the perceived vertical orientation of a visual line with a shift in the internal representation of the gravity vector^9,13^. Several subsequent studies have observed a roll-angle dependency of the optokinetic-induced bias, which is consistent with earlier observations^35,37^. According to the gravito-inertial force resolution hypothesis, integration of the visual rotation signal could induce the estimates of gravity to develop a bias outside the actual otolith signal (gravito-inertial acceleration, GIA)^14^. From the perspective of a static, upright observer, CW visual rotation can be used to infer left ear-down head tilt, causing a rotation of the internal gravitational vector to the CW direction away from the actual otolith signal (GIA)^7,14^; the counterclockwise rotation could induce the opposite. Consistent with this prediction in the rotation of the visual scene, compensatory nystagmus is induced as though the body is being translated to the side^14^. In addition, certain studies have recently evaluated how visual stimuli affect the perceived verticals by varying the angle of the whole body roll tilt. Ward et al. observed an increase in the optokinetic stimuli-induced bias of the SVV with the increasing roll-tilt angle of the head and suggested that visual input was weighted more when the vestibular input became less reliable^11^.

Interestingly, the straight optic flows did not significantly increase the error angles in our data. The test rod placed in the center of the test screen could act as a visual target superimposed on the moving visual background, which suppressed the horizontal optokinetic ocular response^38–40^. However, the test rod could not effectively suppress the background rotational optic flows probably because it was placed in the center of the concentric circular optic flow. In our data, it seems that this type of visual target could not effectively suppress the induced torsional optokinetic ocular responses. However, still more data on the suppression of the torsional optokinetic ocular response induced by rotational visual stimuli are warranted, since the torsional pursuit system has not been clearly demonstrated^41^.

### Pupil changes and cognition

The pupil responds to arousal and mental activity because pupil dilation is correlated with the activity of the noradrenergic locus coeruleus (LC)^42^, a small nucleus in the brainstem that plays a pivotal role in physiological arousal and the regulation of cognitive function^43^. The relationship between the pupillary system and LC-norepinephrine activity has been established through numerous anatomical and physiological studies in humans and animals^44^. Pupil responses have been used extensively to measure the required cognitive load or attention in a variety of tasks^18–21,44–47^. In general, pupil dilation can be promptly observed following the onset of relevant stimuli with an increase in the cognitive demands of the task^18–21^.

The pupil diameter is involuntarily altered by cognitive and autonomic activity^44^. The major pupil responses are induced by luminance changes, which were strictly controlled as shown in the result^48^. Other miscellaneous factors include wakefullness, visual focusing, or anxiety level, which were also largely controlled by the experimental protocols and programs. Therefore, the measured pupil changes could mostly reflect the changes in cognitive activity. Another evidence from our data could be that the pupil responses were different according to the different levels of the task, especially by the velocity of the rotational optic flows. The rotational optic flows of higher velocity, which became more difficult tasks for the participants resulting in higher error angles, and VAS induced larger pupil dilation. Other influencing factors for pupil changes were not altered by this condition.

### Error angles and pupil responses

The error angles of SVV under various visual stimuli are calculated with a fairly complex computational process by integrating multisensory inputs, and multiple brain regions are involved in these neural representations including the cerebellum, brainstem, and several cortical areas such as the putative human ventral intraparietal area^49–51^. We found that the pupil changes were significantly larger under rotational visual stimuli along with increased error angles. When analyzed based on the entire data, the error angle and pupil response were significantly correlated. Error angles in the SVV test, which could reflect the difficulty of verticality recognition, increased with pupil dilation. Considering that there is little change in other factors that can affect the size of the pupil, including luminance, other than the difficulty of the task, these results could suggest an increased demand of cognitive resources to integrate more complex or confusing visual inputs in calculating internal verticality. The larger error angles recorded in the process with larger pupil changes could suggest an increased chance of failed integration when the process demanded higher cognitive resources. The significant correlation between the pupil changes and the VAS reflecting the subjective difficulty of the task also supports the assumption (Fig. 4D).

It is more natural that the pupil changes would be small in static conditions with motionless background objects compared to the conditions in which the background object constituted visual flows. However, the pupil changes under the straight optic flow with a 5°/s velocity were even smaller than those under the preceding static screen, although the difference was not significant (Table 2A). It could indicate that the participants had not yet become sufficiently familiar with the task, despite several rounds of exercise. In other words, the pupil changes under the static screen would reflect the increased cognitive demand from the interpretation of an abruptly encountered unfamiliar task. Hence, the pupil changes under straight 5°/s were also used for the comparison with the pupil changes in the following tasks (Table 2B). Likewise, the error angles were also compared with the error angles under straight 5°/s (Table 1B).

Participants were asked to align the test rod from both the initial tilt offset of 30° (CCW adjustment of the test rod) and 330° (CW adjustment of the test rod) to the direction of the perceived vertical. For rotational flows, the error angles and pupil changes were larger when the test rod was aligned in the opposite direction (tilt offset of 30° under CW rotatory stimuli and tilt offset of 330° under CCW rotatory stimuli) of the optic flows than in the same direction (tilt offset of 30° under CCW rotatory stimuli and tilt offset of 330° under CW rotatory stimuli) (Supplementary Table 1B and 2B). The optokinetic flow in the direction opposite to that of the SVV might serve as a more confusing stimulus and induced an illusionary requirement for a larger adjustment angle with a chance of greater noise accumulation.

### Postural sways

The postural sway was slightly larger under higher velocity rotational flows; however, there was no significant statistical difference for the types of optic flows. Previous studies have revealed that visual stimuli could affect postural stability by providing an excitatory input on the postural muscles^52–54^. The foveal and random optic flows induced larger postural sway with higher variability than peripheral flows and the provoked sways were greater in patients with an impaired retinal function^54,55^. Our condition evaluated a static postural sway in an upright position on a stable-support surface. The tasks modulated visual cues interfering with the otolith organ function leaving vestibular and proprioceptive inputs intact. Therefore, the induced postural reflex might not cause significant sways in the CoG measured in this study. The postural sway of our data could not be comparable to that of the above-mentioned studies since the presented visual stimuli were different in nature. In our study, the centrally placed test bar could induce visual fixation suppressing the optokinetic effect of the visual stimuli.

### Limitations

Healthy adult volunteers without any neuro-otologic and visual disorders were enrolled in this study. The participants’ age ranged from 24 to 63 years including two in their 60 s and majority in their 20–40 s (83.3%, 25/30). There was a positive correlation between age and the error angle (*p* = 0.001, r = 0.186); however, no significant difference was found among groups when comparing the mean error angle by the age group (20 s, 30 s, 40 s, 50 s, and 60 s) probably owing to the small number of participants in their 50 s and 60 s. Pupil change did not show any correlation with age. The heterogeneity of age could be a limitation of this study, and meticulous care is warranted in the interpretation of the data; however, the resultant deviation could be limited considering the small number of the elderly participants. There was no significant difference in the error angle between male and female under most of the conditions, with the exception of a 10°/s rotational optic flow (male, 5.39 ± 2.94; female, 8.26 ± 5.46; *p* = 0.011*). Pupil changes also did not differ by gender under all kinds of optic flow.

The straight optic flow could act as a visual cue aiding in the prediction of the direction of gravitational vertical considering that it is directed linearly forming a fixed angle with the gravitational vector, which could be preferentially applied for the vertical and horizontal optic flows. However, additional physiologic mechanisms are still suggested considering that the error angles were also very small even in the oblique flow (data not shown), which necessitates a more complex internal calculation to estimate the vertical direction.

Pupil response has been interpreted in conjunction with the cognitive activity in a variety of neuro-cognitive research. For its application in measuring the cognitive activity in the visuo-vestibular interactions, we strictly controlled the potential factors affecting the pupil changes, including luminance, wakefulness, consuming drugs, and attention. However, still, caution needs to be exercised in interpreting the clinical meanings of pupil changes in relation with the cognitive functions.

The randomized preset angle of the SVV could have contributed in the reduction of a potential studying effect from repeated measurement. However, it was not applied to the actual measurement considering the resultant variance on the concentration and cognition and to reduce the total test sessions in order to allow the participants to maintain a consistent cognitive function. Randomization needs to be considered in future studies.

This study confirmed that rotational optic flows increase the error angles of the SVV, compared to those under straight optic flows. Larger pupil changes were also confirmed under rotational optic flows potentially suggesting increased cognitive demand. Based on these findings, we could assume that the process of calculating the internal estimates of verticality requires a considerable amount of cognitive resources, especially when confusing visual inputs are presented.

## Supplementary Information

Supplementary Information.
