# Supplementary material for: Pupil responses associated with the perception of gravitational vertical under directional optic flows
Source: Sci Rep. 2021 Oct 29;11:21303. doi: 10.1038/s41598-021-00346-y (PMC8556311; doi:10.1038/s41598-021-00346-y)
Supplement: Supplementary file 1 — Supplementary Information. [file 41598_2021_346_MOESM1_ESM.docx]

**Pupil Responses Associated with the Perception of Gravitational Vertical under Directional Optic Flows**

Running Head: Pupil Responses in Perceiving Gravitational Vertical under Optic Flows

Joo Hyun Park, MD, PhD^1^, Sung Ik Cho, PhD^2^, June Choi, MD, PhD^3^, JungHyun Han, PhD^2^, Yoon Chan Rah, MD, PhD^3^

^1^Department of Otorhinolaryngology-Head and Neck Surgery, Dongguk University College of Medicine; ^2^Department of Computer Science and Engineering, Korea University College of Informatics; ^3^Department of Otorhinolaryngology-Head and Neck Surgery, Korea University College of Medicine, Republic of Korea

*Corresponding Author

Yoon Chan Rah, MD, PhD

Department of Otorhinolaryngology-Head and Neck Surgery,

Korea University Ansan Hospital, Korea University College of Medicine,

123 Jeokgeum-ro, Danwon-gu, Ansan, Gyeonggi-do, 15355, Republic of Korea

Tel: 031-412-4849

Fax: 031-412-5174

E-mail: [ycrah@naver.com](mailto:ycrah@naver.com)

**Supplementary Material**

**Supplementary Figure 1. Postural sways measured by inertia measurement unit (IMU)**

Error bar: mean with 95% confidence intervals

**Supplementary Table 1. Comparison of the error angles for each experimental condition of the optic flows**

**A. Comparison with the error angles of the static background screen**

| Direction 1 | Direction 2 | Velocity  (º/sec) | Error angles (º) | *p*-value |
| --- | --- | --- | --- | --- |
| Static  1.32 ± 1.27º | Horizontal | 5 | 1.38 ± 1.20 | 0.757 |
|  |  | 10 | 1.40 ± 1.49 | 0.691 |
|  | Vertical | 5 | 1.70 ± 1.91 | 0.138 |
|  |  | 10 | 1.70 ± 1.48 | 0.083 |
|  | Straight^†^ | 5 | 1.54 ±1.60 | 0.162 |
|  |  | 10 | 1.55 ±1.49 | 0.116 |
|  | Rotational | 5 | 5.42 ±3.37 | <0.001* |
|  |  | 10 | 7.52 ±5.08 | <0.001* |
|  |  | 50 | 9.49 ± 5.54 | <0.001* |

^†^ Calculated by adding the error angles of horizontal and vertical optic flows

**B. Comparison with the error angles of the straight 5 °/s (horizontal 5 °/s + vertical 5 °/s)**

| Direction 1 | Direction 2 | Velocity  (º/sec) | Error angles (º) | *p-*value |
| --- | --- | --- | --- | --- |
| Horizontal 5º/s  1.38 ±1.20º | Horizontal | 5 | 1.38 ±1.20 º - | - |
|  |  | 10 | 1.40 ± 1.49 | 0.867 |
|  | Vertical | 5 | 1.70 ± 1.91 | 0.100 |
|  |  | 10 | 1.70 ± 1.48 | 0.080 |
|  | Rotation  Opposite direction^†^ | 5 | 5.64 ± 3.46 | <0.001* |
|  |  | 10 | 8.55 ± 5.60 | <0.001* |
|  |  | 50 | 9.56 ± 6.25 | <0.001* |
|  | Rotation  Same direction^‡^ | 5 | 5.21 ± 3.28 | <0.001* |
|  |  | 10 | 6.49 ± 4.29 | <0.001* |

^†^ Indicating that the direction of the testing rod adjustment and the background rotational optic flow were opposite.

^‡^ Indicating that the direction of the testing rod adjustment and the background optic flow were the same.

**Supplementary Table 2. Comparison of pupil changes for each experimental condition of optic flows**

**A. Comparison with the pupil changes of the static background screen**

| Direction 1 | Direction 2 | Velocity  (º/sec) | Pupil changes  (mm) | *p*-value |
| --- | --- | --- | --- | --- |
| Static  Pupil changes  (mm)  0.77 ± 0.36 | Horizontal | 5 | 0.74 ± 0.27 | 0.411 |
|  |  | 10 | 0.73 ± 0.32 | 0.358 |
|  | Vertical | 5 | 0.70 ± 0.32 | 0.206 |
|  |  | 10 | 0.75 ± 0.32 | 0.678 |
|  | Straight^†^ | 5 | 0.72 ± 0.30 | 0.132 |
|  |  | 10 | 0.74 ± 0.32 | 0.323 |
|  | Rotational | 5 | 0.89 ± 0.38 | 0.007* |
|  |  | 10 | 0.68 ± 0.23 | 0.008* |
|  |  | 50 | 0.98 ± 0.58 | 0.019* |

^†^ Calculated by adding the error angles of horizontal and vertical optic flows

**B. Comparison with the pupil changes of the** **straight 5 °/s (horizontal 5 °/s + vertical 5 °/s)**

| Direction 1 | Direction 2 | Velocity  (º/s) | Pupil changes  (mm) | *p*-value |
| --- | --- | --- | --- | --- |
| Horizontal 5 º/s  0.74 ± .27 | Horizontal | 5 | - | - |
|  |  | 10 | 0.73 ± 0.32 | 0.743 |
|  | Vertical | 5 | 0.70 ± 0.32 | 0.487 |
|  |  | 10 | 0.75 ± 0.32 | 0.587 |
|  | Rotation  Opposite direction^†^ | 5 | 0.94 ± 0.42 | 0.030* |
|  |  | 10 | 0.88 ± 0.36 | 0.061 |
|  |  | 50 | 0.95 ± 0.55 | 0.023* |
|  | Rotation  Same direction^‡^ | 5 | 0.85 ± 0.33 | 0.188 |
|  |  | 10 | 0.86 ± 0.39 | 0.239 |

^†^ Indicating that the direction of the testing rod adjustment and the background rotational optic flow were opposite.

^‡^ Indicating that the direction of the testing rod adjustment and the background optic flow were the same.

**Supplementary Table 3. Average illuminance of the test screen according to the type of background optic flow**

|  | Y unit of YIQ color space | Lightness value (L) of CIE-Lab |
| --- | --- | --- |
| Horizontal 5 º/s | 0.06630 ± 0.00107 | 5.08 ± 0.10 |
| Horizontal 10 º/s | 0.06683 ± 0.00122 | 5.09 ± 0.11 |
| Vertical 5 º/s | 0.06688 ± 0.00154 | 5.14 ± 0.15 |
| Vertical 10 º/s | 0.06601 ± 0.00109 | 5.08 ± 0.11 |
| CW 5º/s | 0.06762 ± 0.00126 | 5.19 ± 0.12 |
| CW 10º/s | 0.06603 ± 0.00128 | 5.09 ± 0.12 |
| CW 50º/s | 0.06704 ± 0.00197 | 5.16 ± 0.15 |
| CCW 5º/s | 0.06667 ± 0.00106 | 5.12 ± 0.11 |
| CCW 10 º/s | 0.06607 ± 0.00084 | 5.06 ± 0.09 |
| CCW 50 º/s | 0.06697 ± 0.00127 | 5.13 ± 0.12 |
| *p*-value | 0.293 | 0.367 |

CW, clockwise; CCW, counter-clockwise
